# Buccolingual Inclination of Second Molars in Untreated Adolescents and Adults with Near Normal Occlusion: A CBCT Study

**DOI:** 10.3390/jcm11226629

**Published:** 2022-11-08

**Authors:** Chenshuang Li, Boryana Dimitrova, Normand S. Boucher, Chun-Hsi Chung

**Affiliations:** Department of Orthodontics, School of Dental Medicine, University of Pennsylvania, Philadelphia, PA 19104, USA

**Keywords:** buccolingual inclination, CBCT, second molars, adolescents, adults

## Abstract

The mandibular second molars are lingually positioned relative to the alveolar ridge and have a limited amount of lingual alveolar bony support. As the maxillary second molars are articulated with the mandibular second molars, maintaining the normal buccolingual inclination of both maxillary and mandibular second molars would potentially help to not only optimize the masticatory function, but also avoid dehiscence and fenestration. The current study evaluated the buccolingual inclination of second molars in untreated adolescents and adults. One hundred and two Caucasian subjects with skeletal class I and minimum dental arch crowding/spacing were selected and divided into two groups: (1) adolescent group: age 12–18 years, N = 51 (21 females, 30 males); (2) adult group: age 19–65 years, N = 51 (40 females, 11 males). For each subject, the inclination for each second molar was measured as the angle between the long axis of each tooth and a vertical line on cone beam computed tomography images. The Mann–Whitney *U* test was used for intergroup comparisons. Maxillary second molars exhibited a buccal inclination of 15.30° in the adolescent group and 15.70° in the adult group. Mandibular second molars exhibited a lingual inclination of 17.05° in the adolescent group and 15.20° in the adult group. No statistically significant differences were detected between the age groups. In addition, a statistically significant difference was only found between genders in the adolescent group for the maxillary second molar inclination. In summary, maxillary second molars exhibited buccal inclination and mandibular second molars exhibited lingual inclination. The amount of buccolingual inclination of the second molars was similar in the adolescent and adult groups.

## 1. Introduction

As early as 1911, Wilson [1] described a curve that, when viewed from the front, contacts the buccal and lingual cusps of the maxillary and mandibular molars and is lower in the middle. This curve is known today as the “curve of Wilson”. It was hypothesized that such a naturally occurring curve allows the buccolingual inclination of the posterior teeth to be parallel with the inward pull and orientation of the medial pterygoid muscle contraction [2]. It not only produces the greatest resistance to masticatory forces [2] and provides open access to the bolus of food for sufficient chewing efficiency while ensuring the most effective use of cuspal contacts [2,3], but also helps avoid nonfunctional contacts [3].

The existence of a buccolingual inclination of the posterior teeth is acknowledged by the American Board of Orthodontics [4], where the discrepancy between buccal and lingual cusps of the maxillary and mandibular posterior teeth should be within 1 mm after orthodontic treatment. To provide guidance for clinical evaluations, various studies have evaluated the buccolingual inclinations of molar crowns by utilizing dental models [5,6]. However, due to the variations in crown morphology from tooth development, restorations, and wear and tear, there are limitations in evaluating tooth inclination by using dental casts [7].

With the broad adaption of cone beam computed tomography (CBCT) in the orthodontic field in the past decades [8], practitioners can now visualize and evaluate not only the buccolingual inclination of a posterior tooth by using the true long axis bisecting both the crown and the roots of the tooth [9,10,11,12], but also the root position in relation to the alveolar ridge [13,14,15], which provides more detailed information about the biologic boundary of orthodontic tooth movement, especially in the transverse dimension.

In fact, periodontal complications have been reported when teeth are moved orthodontically beyond the bony housing [16]. It is also worth noting that the root contact with the cortical plate at the level of the root apex during orthodontic tooth movement has been associated with root resorption [17]. Thus, maintaining the tooth roots in the bony housing is important while setting the orthodontic treatment objectives and performing active orthodontic tooth movements. Interestingly, strong attention has been paid to the buccolingual inclination of the first molars [5,6,9,10,11,12,15,18,19,20,21,22], but less to that of the second molars [11,12,23]. However, when comparing the root positions of the molars to the alveolar ridge and bony body of the mandible, differences were noticed. The mandibular first molars are positioned in the relatively central position of the alveolar ridge and mandibular body, while the mandibular second molars are lingually positioned with a significant lingually inclined alveolar ridge relative to the mandibular body. This forms a deeper lingual concavity in the mandible under the second molars [24]. In addition, mandibular first molars have relatively even thicknesses of the buccal and lingual alveolar bone, while the mandibular second molars have thicker buccal alveolar bone and thinner lingual alveolar bone [13,14]. The thicker buccal alveolar bone of the mandibular second molar corresponds to the oblique ridge, which provides sounder bone for temporary anchorage devices (TADs) [25]. On the other hand, the thinner lingual alveolar bone of the mandibular second molar may define the anatomic limit for orthodontic tooth movement [26] and be subject to a higher risk of dehiscence and fenestration when inappropriate orthodontic treatment is implemented. Thus, a proper buccolingual inclination of the mandibular second molars is even more crucial to avoid periodontal complications during orthodontic treatment.

Previously, several studies [11,12,21,23] reported buccal inclination of the maxillary second molars and lingual inclination of the mandibular second molars by utilizing CBCT images, but the inclination degrees are not the same. Tong et al. [23] included 76 untreated subjects, ranging from 12 to 36 years old, with a population of mixed ethnicity, and reported that the maxillary second molars had 10.83° ± 4.97° buccal inclination and the mandibular second molars had 12.38° ± 4.92° lingual inclination. Kasai and Kawamura [12] reported that the mandibular second molars had an average lingual inclination of 21.1° in modern Japanese adults (age range: 18 years to 48 years). Barrera et al. [11] showed an average of 12.5° buccal inclination of maxillary second molars in 10 adult subjects with normal occlusion, while a recent study conducted by Golshah et al. stated that the maxillary second molars had 13.05° ± 4.91° buccal inclination and the mandibular second molars had 16.44° ± 5.28° lingual inclination, and the molar inclination varies among the patients with different sagittal skeletal patterns [21]. The differences in the amount of molar inclination among the studies might be due to the variations in age, since the self-uprighting with growth observed in the first molars [6,9] may also apply to the second molars. In addition, the different ethnic backgrounds of the subjects in each study may also contribute to the data variation.

Thus, by utilizing a large CBCT database, the current study aims to evaluate the buccolingual inclination of maxillary and mandibular second molars in untreated Caucasian adolescents and adults with near normal occlusion, which could potentially help to optimize the clinical diagnosis and guide the personalized orthodontic care.

## 2. Materials and Methods

The study was conducted in accordance with the Declaration of Helsinki and approved by the Institutional Review Board of the University of Pennsylvania (protocol #829468). The study is a retrospective study utilizing the pre-existing CBCT images taken in private practice with an I-CAT machine (Images Sciences International, Hatfield, PA, USA) with 0.300 mm voxel size. The CBCT images of Caucasian subjects with an age between 12 to 65 years old were screened. The inclusion criteria were: (1) no prior orthodontic treatment, (2) fully erupted maxillary and mandibular second molars and completely formed roots, (3) skeletal Class I (ANB angle 0–4°), (4) minimal dental wear, (5) less than 5 mm of crowding per arch and less than 5 mm of spacing per arch, and (6) no missing teeth other than third molars. Subjects were excluded for the following reasons: (1) posterior crossbite, (2) crowns or significant restorations on any second molar, (3) the presence of primary or supernumerary teeth, or (4) craniofacial deformities or evident facial or skeletal asymmetry. All the subjects who met the inclusive and exclusive criteria were included for further analysis.

### 2.1. Sample Size Calculation

The sample size was calculated with G*Power (Version 3.1.9.4) [27]. To ensure an adequate sample size for statistical differences, 18 teeth (minimum nine patients, including left and right sides) were needed for each group based on a power analysis with α = 0.05 and 80% power with an effect size of 1.0. Comparisons were conducted between genders and between two age groups (adolescent group (age 12–18 years) and adult group (age 19–65 years)).

### 2.2. Imaging Analysis

**Figure 1:**
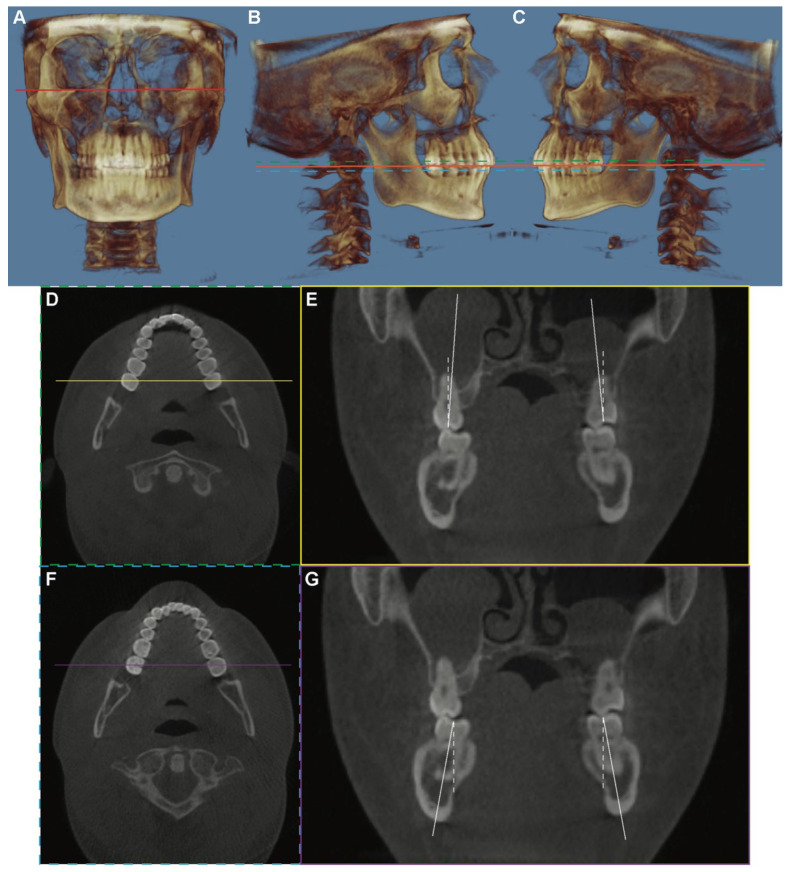
The demography of measurement workflow. (**A**) The CBCT image was oriented such that a line connecting the inferior border of the orbital rims (red line) was parallel to the floor. (**B**,**C**) The CBCT image was oriented such that the functional occlusal plane (orange line) was parallel to the floor. The axial sections of the maxillary arch (green dot line) and the mandibular arch (blue dot line) were used for sagittal guideline determination. (**D**,**E**) The measurement of the buccolingual inclination of the maxillary second molars. (**D**) On the axial section of the maxillary arch, the location of the coronal slide was defined as a line (yellow line) that passes through the midpoint of the mesiodistal occlusal crown width of bilateral second molars. (**E**) The coronal cross section was obtained in a 0.5 mm slice. The long axis of the tooth (solid white line) was defined as a line connecting the midpoint of the buccal and lingual cusp tips and the midpoint of the buccolingual width at the cervical base close to the furcation of the anatomic crown. The angle at which each molar’s long axis intersects a true vertical line (white dot line) was measured and represents the inclination of the tooth. (**F**,**G**) The measurement of the buccolingual inclination of the mandibular second molars. The axial section of the mandibular arch was used. The purple line represented the location of the coronal slide, as shown in (**G**).

The CBCT DICOM files were imported into Dolphin 3D software (Dolphin Imaging; version 11.95 Premium, Chatsworth, CA, USA) and oriented such that the functional occlusal plane and a line connecting the inferior border of the orbital rims were parallel to the floor (Figure 1). For the maxillary or mandibular arch, the coronal cross section was obtained in a 0.5 mm slice using a section that best fit the bilateral second molars’ mesiodistal midpoints. The long axis of each tooth was defined as a line connecting the midpoint of the buccal and lingual cusp tips and the midpoint of the buccolingual width at the cervical base close to the furcation of the anatomic crown. The angle at which each molar’s long axis intersects a true vertical line, perpendicular to the horizontal reference plane, was measured and represents the inclination of the tooth. If the crown was lingual to the roots, the inclination would be negative, and if it was buccal to the roots, the inclination would be positive.

### 2.3. Statistical Analysis

All measurements were performed on both the left and right sides of each sample and were taken by the same examiner. Twenty samples (10 in the adolescent group and 10 in the adult group) were randomly selected and remeasured at a 1-week interval by the same examiner. A paired *t*-test was used to determine whether there were significant differences between the original and repeated tooth inclination measurements. The Shapiro–Wilk normality test was performed using OriginPro 8 (Origin Lab Corp., Northampton, MA, USA). Some data did not follow the normal distribution, so all data are presented as median with range (Min, Max). A Chi-Square analysis was performed to compare the distribution of subjects in each gender and age group. For intergroup comparisons of the age or molar inclination, the Mann–Whitney *U* test was used. For all data presented in this manuscript, *p* < 0.05 was considered a statistically significant difference.

## 3. Results

### 3.1. Patient Demographic Information

The intra-examiner test showed no significant differences in repeated measurements. The Pearson correlation coefficients varied between 0.95 and 0.99 for the measurements.

**Figure 2:**
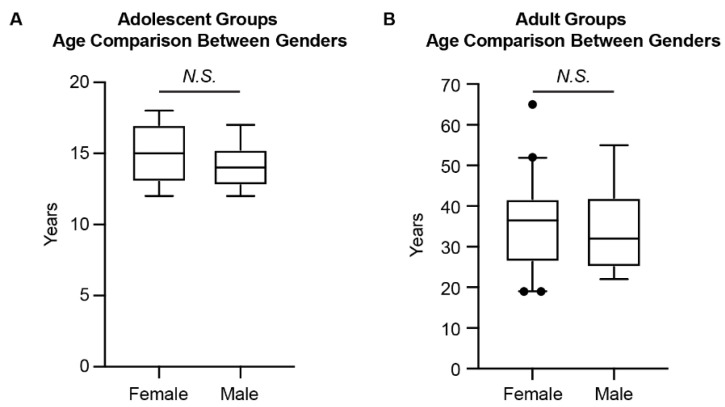
The age comparison between genders for the adolescent and adult groups. (**A**) The age comparison for the adolescent groups. (**B**) The age comparison for the adult groups. The Mann–Whitney *U* test was used. The box plot represents the 5th percentile, 25th percentile, median (50th percentile), 75th percentile, and 95th percentile, respectively. N.S.: not significant.

After screening the CBCT datasets as described above, there were 51 subjects (21 females and 30 males) in the adolescent group (age 12–18 years) and 51 subjects (40 females, 11 males) in the adult group (age 19–65 years) (Table 1, Figure 2). For the sample population included in the current study (Table 1, Figure 2), there was no significant difference in age between females and males for both the adolescent and adult groups. However, the Chi-Square analysis showed the difference in gender distribution between the two age groups. Thus, comparisons between age groups and genders, respectively, were performed in the following data analysis.

### 3.2. Differences in the Inclinations of the Second Molars between Genders

**Figure 3:**
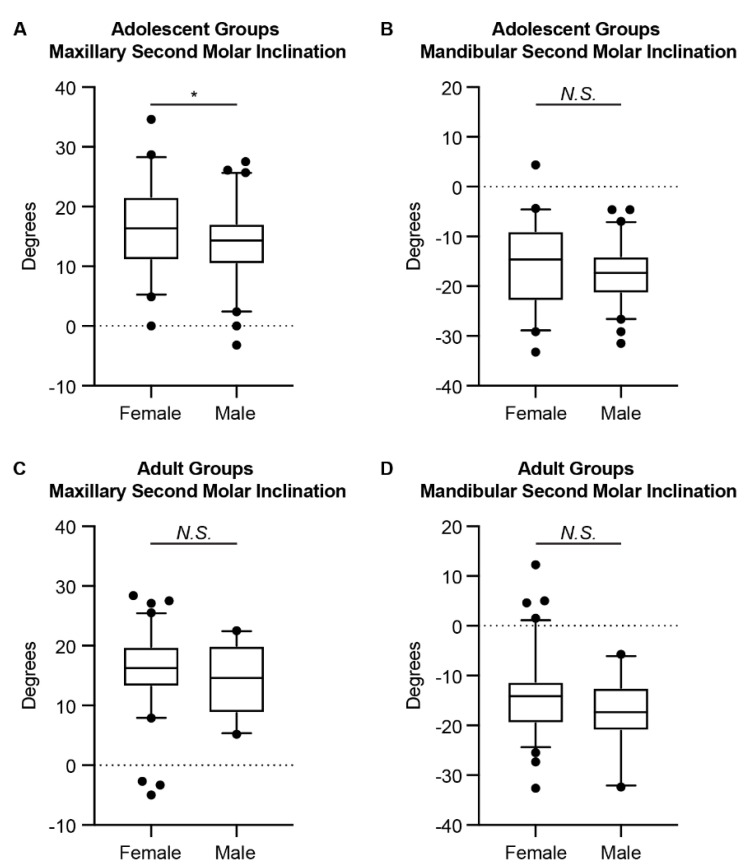
The comparison of the second molar inclination between genders. The (**A**) maxillary and (**B**) mandibular second molar inclination of females and males in the adolescent groups. The (**C**) maxillary and (**D**) mandibular second molar inclination of females and males in the adolescent groups. The Mann–Whitney *U* test was used. The box plot represents the 5th percentile, 25th percentile, median (50th percentile), 75th percentile, and 95th percentile, respectively. *: *p* < 0.05; N.S.: not significant.

In all groups, the maxillary second molar showed buccal inclination, and the mandibular second molar showed lingual inclination (Table 2, Figure 3). When comparing between genders, a statistically significant difference was only detected for the maxillary second molars in the adolescent group. However, a trend was detected for the mandibular second molars in the adolescent group (*p*-value = 0.0928). The adult group detected no statistically significant difference between genders for the maxillary and the mandibular second molars.

### 3.3. Differences in the Inclinations of the Second Molars between Adolescents and Adults

**Figure 4:**
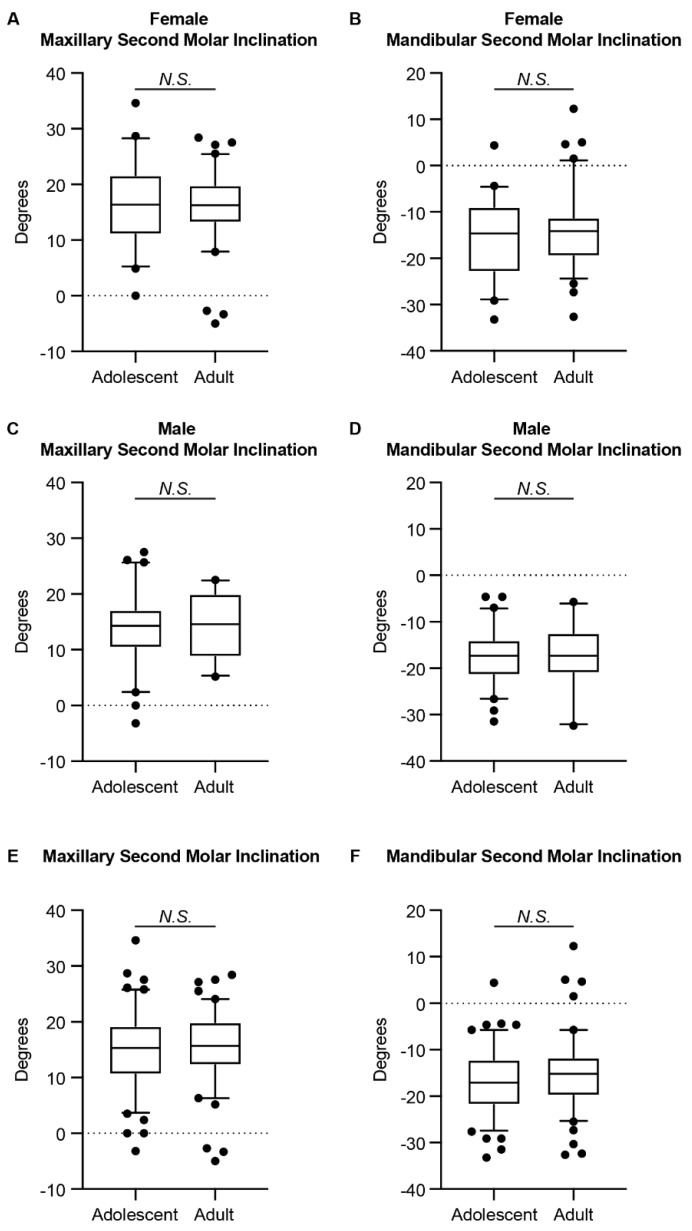
The comparison of the second molar inclination between age groups. The (**A**) maxillary and (**B**) mandibular second molar inclination of females in the adolescent and adult groups. The (**C**) maxillary and (**D**) mandibular second molar inclination of males in the adolescent and adult groups. The (**E**) maxillary and (**F**) mandibular second molar inclination of both genders in the adolescent and adult groups. The Mann–Whitney *U* test was used. The box plot represents the 5th percentile, 25th percentile, median (50th percentile), 75th percentile, and 95th percentile, respectively. N.S.: not significant.

Previous studies reported the differences in the inclinations of the first molars among different age groups [6,9]. To evaluate if self-uprighting with growth also occurs with the second molars, we further compared the inclinations of the second molars between adolescents and adults. As shown in Table 3 and Figure 4, no statistically significant difference was detected for the second molar inclination between age groups.

## 4. Discussion

A major advantage of using CBCT to determine the long axis of molars is to avoid biases associated with tooth morphology or uneven wear of the cusps [19,23]. In a manner similar to Alkhatib and Chung [10] and Barrera et al. [11], we measured the long axis of the molars by using a line connecting the midpoint of the buccal and lingual cusp tips and the midpoint of the buccolingual width at the cervical base, close to the furcation of the anatomic crown. This provides a reliable way to determine the long axes for the maxillary and mandibular molars, since the dilacerations of the roots were not factored in. The consistency of the two-time measurements further validated the reliability of the current measurement protocol.

Overall, the maxillary second molar showed buccal inclination, and the mandibular second molar showed lingual inclination, which is consistent with previous studies [11,12,21,23]. However, when comparing the degree of inclinations in detail, differences were represented. In the current study with all Caucasian subjects, we found that the median value of buccal inclination of the maxillary second molars in untreated adolescents was 15.30°, and in untreated adults this was 15.70°. These values are slightly larger than the values reported previously by Tong et al. (10.83°) [23], Barrera et al. (12.45°) [11], and Golshah et al. (13.05°) [21]. For the lingual inclination of the mandibular second molars, we found that the median value was 17.05° in untreated adolescents and 15.20° in untreated adults, which are slightly larger than the values reported previously by Tong et al. (12.38°) [23], similar to the values reported by Golshah et al. (16.44°) [21], but smaller than the value reported by Kasai and Kawamura (21.1°) [12]. As the studies involved subjects of various ethnic backgrounds, these differences in molar inclination indicated that, similar to cephalometric tracing values, the normative values of molar inclination should be established for different populations.

When comparing the second molar inclination between genders, we detected a statistically significant difference for the maxillary second molars in the adolescent group and a tendency for the mandibular second molars in the adolescent group, with female adolescents having greater maxillary second molar buccal inclination and less mandibular second molar inclination than male adolescents. This is different from what was reported previously with first molars in that no difference was detected between genders for adolescents [9]. For the adult group, no difference was detected between genders for second molar inclinations, which is the same as reported previously with first molars [9,10]. The different gender-related trends of first and second molar inclinations may be related to the gender-related difference in maxillary and mandibular alveolar morphology [28], as well as the maxilla and mandible growth and development [29] and shape [30,31], since the second molars are positioned at the distal end of the alveolar ridge. In addition, the gender difference detected in the adolescent group might be correlated to the timing of transverse growth in males and females, as the transverse growth in females stops earlier than in males [32]. Further studies are needed to investigate the gender-related difference in the posterior alveolar ridge development and remodeling during growth and development, as well as during aging.

When comparing the second molar inclination between adolescents and adults, we did not find any significant difference for both the maxillary and the mandibular second molars. Thus, unlike the first molars [6,9], it seems that the self-uprighting with growth does not occur on the second molars. One possible explanation is that the maxillary and mandibular arches have completed most of their transverse growth by the time the second molars erupt, as indicated by Moyers et al. [33]. In addition, both the maxillary and mandibular second molars showed greater inclination than the first molars [6,9]. This trend is the same as previous studies [11,12,23]. The progressive inclination of the molars is coincidental with the alveolar bone inclination, especially for the mandible [24]. Nevertheless, the tongue is a significant oral squamous cell carcinoma (OSCC) site, especially in adolescents and young adults [34]. Since the mandibular second molar usually touches the tongue, the position of the second molar might be a critical clinicopathological factor of oral tongue squamous cell carcinoma (OTSCC). In fact, Kim et al. reported that the abnormally lingually inclined position of the mandibular second molar and following narrow tongue space are associated with OTSCC development in young mature patients [35]. Thus, our results agree with previous studies [2,3,36], in that a proper curve of Wilson should be maintained after orthodontic treatment—especially at the level of the second molars—to allow for effective intercuspation while avoiding nonfunctional contacts known as balancing interferences, in order to maintain sufficient alveolar bony support around the teeth and avoid the potential risk of OTSCC.

This study does have some limitations that must be considered before accepting its conclusions. First, the comparison between different age groups is a cross-sectional study. There is no doubt that a longitudinal study would provide a stronger level of evidence. However, taking several CBCT images on the same patient without treatment needs is unacceptable and unethical. Secondly, to make our data comparable with the previous publications, we only recorded the chronological age of the included subjects. It is worth noting that as chronological age, skeletal age, and dental age do not necessarily equal each other in each individual, skeletal age is an important factor to be considered when evaluating growth and development [37,38], as the tooth position and angulation would adapt to the bony structure growth and development. This limitation may explain the data variation observed in each group. Thirdly, although the number of included subjects meets the sample size requirements, the male adult group had a relatively smaller sample size compared to the other three groups due to the subjects available from the database we used. Further studies with a larger sample size from multiple clinics might be considered. Lastly, the relationship between the buccolingual inclination of molars and vertical facial type has been studied, but the results have not been consistent [18,20]. In the current study, we did not compare the molar inclinations among groups with different vertical facial types. Further study is warranted to determine if a relationship exists.

## 5. Conclusions

The following conclusions can be drawn from this study:

- Maxillary second molars exhibited a buccal inclination of 15.30° in the Caucasian adolescent group and 15.70° in the Caucasian adult group.
- Mandibular second molars exhibited a lingual inclination of 17.05° in the Caucasian adolescent group and 15.20° in the Caucasian adult group.
- Female adolescents had more buccal inclination of the maxillary second molars and less lingual inclination of the mandibular second molars than male adolescents. There was no difference in the second molar inclinations between genders in adults.
- The amount of inclination of the second molars was similar in adolescent and adult groups.
- Clinically, a proper buccolingual inclination of the upper and lower second molars should be maintained.

## Figures and Tables

**Table 1:** The characters of the subjects included in the current study.

	Female			Male			Mann–Whitney *U* Test *p*-Value
	N	Median	Range (Min, Max)	N	Median	Range (Min, Max)	
Adolescent (12–18 years)	21	15.00	(12, 18)	30	14.00	(12, 17)	0.0525
Adult (19–65 years)	40	36.50	(19, 65)	11	32.00	(22, 55)	0.8081
	*p*-Value of Chi-Square Analysis for Gender Distribution						0.0001

**Table 2:** The comparison of the second molar inclination between genders. U7s: maxillary second molars; L7s: mandibular second molars.

		Female			Male			Mann–Whitney *U* Test *p*-Value
		N	Median	Range (Min, Max)	N	Median	Range (Min, Max)	
Adolescent (12–18 years)	U7s	42	16.35	(0.00, 34.60)	60	14.30	(−3.20, 27.50)	0.0354
	L7s	42	−14.65	(−33.20, 4.40)	60	−17.30	(−31.50, −4.60)	0.0928
Adult (19–65 years)	U7s	80	16.25	(−5,00, 28.40)	22	14.60	(5.20, 22.50)	0.1621
	L7s	80	−14.10	(−32.60, 12.30)	22	−17.30	(−32.40, −5.70)	0.3615

**Table 3:** The comparison of the second molar inclination between age groups. U7s: maxillary second molars; L7s: mandibular second molars.

		Adolescent (12–18 Years)			Adult (19–65 Years)			Mann–Whitney *U* Test *p*-Value
		N	Median	Range (Min, Max)	N	Median	Range (Min, Max)	
Female	U7s	42	16.35	(0.00, 34.60)	80	16.25	(−5,00, 28.40)	0.6376
	L7s	42	−14.65	(−33.20, 4.40)	80	−14.10	(−32.60, 12.30)	0.8458
Male	U7s	60	14.30	(−3.20, 27.50)	22	14.60	(5.20, 22.50)	0.8578
	L7s	60	−17.30	(−31.50, −4.60)	22	−17.30	(−32.40, −5.70)	0.5763
Total	U7s	102	15.30	(−3.20, 34.60)	102	15.70	(-5.00, 28.40)	0.3951
	L7s	102	−17.05	(−33.20, 4.40)	102	−15.20	(−32.60, 12.30)	0.2218

## Data Availability

The data presented in this study are available on request.
